# Characterization of Traumatic Injury During the Early COVID-19 Pandemic: Results From a National Healthcare Database

**DOI:** 10.7759/cureus.28257

**Published:** 2022-08-22

**Authors:** Ashley Sun, Daniel Johnson

**Affiliations:** 1 Department of Emergency Medicine, Penn State College of Medicine, Hershey, USA; 2 Department of Emergency Medicine, Penn State Health Milton S. Hershey Medical Center, Hershey, USA

**Keywords:** coronavirus disease 2019, covid-19, epidemiology and public health, crisis health, traumatic injury, sars-cov-2

## Abstract

Objective: To characterize traumatic injury patterns after stay-at-home orders were implemented in the United States in response to the coronavirus disease 2019 (COVID-19).

Methods: A retrospective review of a convenience sample of patients from a national healthcare research database (TriNetX) was conducted from April 1, 2020, to June 30, 2020. Inclusion criteria included all patients with documentation of both injury pattern and mechanism of injury. A comparison was made to a matched pre-pandemic timeframe. Changes in percentage and rate ratio (RR) with a 95% confidence interval were reported. RRs were calculated using Poisson regression analysis.

Results: A total of 238,661 patients in the control and 178,224 patients in the study cohorts were analyzed. Significant increases in assaults (RR: 1.17, 95% CI: 1.14, 1.20) and bicycle accidents (RR: 1.07, 95% CI: 1.04, 1.11) were noted. There was a relative increase in patients who were male (+1.78%) and white (+2.01%). More injuries were alcohol-related (+0.76%) and occurred at home (+0.79%). A decrease in motor vehicle accidents (-1.17%), foot and ankle injuries (-1.63%), and injuries occurring at sporting events (-0.54%) was noted.

Conclusions: Changes in injury patterns were observed during the study period. During future crises, particular public health and injury prevention resources may be required to address assaults, substance abuse, and home safety.

## Introduction

The first cases of coronavirus disease 2019 (COVID-19), caused by the novel severe acute respiratory syndrome coronavirus 2 (SARS-CoV-2) virus, were reported in late December 2019. By mid-March 2020, the virus had spread worldwide, a pandemic was declared by the World Health Organization, and a national state of emergency was instituted in the United States [1]. The earliest mitigation efforts before the development of vaccinations or wide availability of testing consisted of various forms of physical distancing such as stay-at-home orders. Patterns in healthcare utilization changed as elective procedures were delayed, and patients avoided visits for preventative care and non-urgent needs [2]. Trends in traumatic injury appeared to be affected by the change in the day-to-day life of Americans [3,4].

While it is imperative to understand the direct health effects of the SARS-CoV-2 virus and its clinical syndromes, equal attention is due to the broader effects of both the disease and mitigation effects on public health [5]. Psychosocial stressors, including substance abuse, have also complicated the pandemic [6] and can contribute to violence and other mechanisms of traumatic injury. Understanding the changes to injury patterns is critical to efforts aimed at injury prevention. Prior research on this topic has predominantly been produced by single, large, urban, trauma centers that focus on a distinct geographic area [3,7,8].

The objective of this study is to characterize the types and mechanisms of traumatic injury during the stay-at-home phase of the COVID-19 pandemic with the goal of helping to guide public health policy during future crises. This study broadens the current scope of knowledge by utilizing a national healthcare research database. It was hypothesized that injury pattern, mechanism of injury, location of the injury, and factors associated with injury would vary due to the COVID-19 stay-at-home orders.

## Materials and methods

Study design

This is a retrospective review of a convenience sample of patients from a national, electronic health record research database. The use of such databases has been described in the literature as a means to guide public health policy in real time [9], which is the aim of this study. The data used in this study were collected on October 28, 2021, from the TriNetX research network, which provided access to electronic medical records (diagnoses, procedures, medications, laboratory values, and genomic information) from approximately 50 million patients from 49 healthcare organizations. TriNetX, LLC is compliant with the Health Insurance Portability and Accountability Act (HIPAA), the US federal law that protects the privacy and security of healthcare data, and any additional data privacy regulations applicable to the contributing healthcare organization. TriNetX is certified to the International Organization for Standardization (ISO) 27001:2013 standard and maintains an information security management system (ISMS) to ensure the protection of the healthcare data it has access to and to meet the requirements of the HIPAA Security Rule. Any data displayed on the TriNetX Platform in aggregate form, or any patient-level data provided in a dataset generated by the TriNetX platform, only contain de-identified data as per the de-identification standard defined in Section §164.514(a) of the HIPAA Privacy Rule. The process by which the data are de-identified is attested to through a formal determination by a qualified expert as defined in Section §164.514(b)(1) of the HIPAA Privacy Rule.

Details regarding the TriNetX database have been previously described in the literature [10]. Information regarding patient demographics, injury pattern (body area involvement), mechanism of injury, location of the injury, and factors associated with the injury including alcohol use, abuse, and assaults were obtained utilizing a query of diagnostic codes (International Classification of Diseases, Tenth Revision (ICD-10)). Data gathering and descriptive statistics were conducted using the TriNetX online platform. These datasets are protected from the public and are available for use by research institutions upon request. Given the de-identified nature of the dataset and its compliance with HIPAA security standards, the study was given a determination of non-human subject research by the Human Subject Protection Office and Institutional Review Board of Penn State University (STUDY00017757).

Selection of participants

The TriNetX research database compiles data from a variety of inpatient and outpatient settings. All patients in the database with data pertaining to both a traumatic injury and recorded mechanism of injury were included in the study. For purposes of data obfuscation, the TriNetX platform reports an age of 90 for all patients greater than 89 years old. Two patient cohorts were built using date ranges pertaining to the highest rates of stay-at-home orders in the United States. The post-stay-at-home (study) cohort included patients who presented between April 1, 2020, and June 30, 2020. This three-month period was selected to account for a high likelihood of strict adherence to the mandates as cross-sectional studies demonstrated decreasing adherence toward the end of this period [11]. A pre-stay-at-home (control) cohort included patients who presented over the same date range in 2019. We elected to use the same date range rather than the preceding three-month period to control for seasonal variation in trauma epidemiology.

Data collection and processing

Inclusion criteria and date ranges were input into the platform, and a query was run to build the patient cohort using ICD-10 coding for injury patterns and mechanisms. Through proprietary algorithms, a sample from each healthcare organization included in the research database was obtained in a non-randomized fashion to generate a smaller cohort for analysis. Demographics and injury characteristics were compared independently on the platform, and the size of the cohort generated was based on the number of comparisons. Therefore, a smaller number of patients were included when analyzing injury characteristics versus demographics related to the number of comparisons. For this reason, we described these cohorts as a "convenience" sample. The output of this process included demographics and rates of occurrence of diagnostic codes of the represented patients.

Data analysis

A “compare cohorts” analytic function using the TriNetX online platform compared patient demographics including age, gender, race, and ethnicity. The remainder of the data were produced as rates of occurrence of each diagnostic code. These were compared between the two cohorts using Poisson regression analysis, and a rate ratio (RR) was calculated. For this study, a rate ratio greater than 1 indicates that the rate was greater in the study versus the control. For each RR, a 95% confidence interval and p-value were calculated. The percentage of change between the cohorts was also calculated.

## Results

Between April 1, 2020, and June 30, 2020, a total of 323,576 patients in the control and 200,667 patients in the study cohort were analyzed to describe patient demographics (Table 1). The average age was 43.8 (SD: 24.4) years and 50.5% were males. The study cohort demonstrated a decrease in 122,909 patient presentations (62%). The study cohort saw a higher percentage of patients who were male (52.3% vs. 50.5%) and white (65.2% vs. 67.2%). A corresponding decrease in patients identified as black or African American was noted (-1.56%).

**Table 1:** Demographics and injury characteristics

	April-June 2019		April-June 2020	
	(n = 323,576)		(n = 200,667)	
Demographics				
Age, average (SD)	43.8 (24.4)		45.1 (24.2)	
Male	163,494	50.53%	104,971	52.31%
Female	158,627	49.02%	95,234	47.46%
Unknown	1,458	0.45%	462	0.23%
Ethnicity, n (%)				
Unknown	123,971	38.31%	76,359	38.05%
Not Hispanic or Latino	173,342	53.57%	109,523	54.58%
Hispanic or Latino	26,266	8.12%	14,785	7.37%
Race, n (%)				
Unknown	43,989	13.59%	27,145	13.53%
White	211,095	65.24%	134,940	67.25%
Black or African American	59,321	18.33%	33,663	16.78%
Asian	7,646	2.36%	3,890	1.94%
American Indian or Alaskan Native	756	0.23%	515	0.26%
Native Hawaiian or Pacific Islander	772	0.24%	514	0.26%
Characteristics	(n = 238,661)		(n = 178,224)	
Injury pattern, n (%)				
Head	98,717	41.36%	74,560	41.83%
Neck	43,606	18.27%	31,707	17.79%
Thorax	49,231	20.63%	38,465	21.58%
Abdomen/pelvic	56,331	23.60%	43,346	24.32%
Knee and lower leg	67,022	28.08%	49,452	27.75%
Wrist, hand, fingers	66,685	27.94%	49,274	27.65%
Ankle and foot	56,308	23.59%	39,136	21.96%
Shoulder and upper arm	51,861	21.73%	39,369	22.09%
Elbow and forearm	40,911	17.14%	31,813	17.85%
Multiple trauma	12,789	5.36%	10,317	5.79%
Burns	12,921	5.41%	10,257	5.76%
Mechanism of injury, n (%)				
Fall	97,969	41.05%	72,606	40.74%
Car occupants	24,925	10.44%	15,566	8.73%
Motorcycle accidents	4,224	1.77%	4,056	2.28%
Pedestrian accidents	6,447	2.70%	4,807	2.70%
Bicycle accidents	5,738	2.40%	6,158	3.46%
Intentional self-harm	2,580	1.08%	2,415	1.36%
Assault	11,725	4.91%	13,684	7.68%
Physical abuse, n (%)				
Adult, suspected	481	0.20%	366	0.21%
Adult, confirmed	851	0.36%	501	0.28%
Child, suspected	343	0.14%	254	0.14%
Child, confirmed	238	0.10%	136	0.08%
Alcohol associated, n (%)	4,642	1.95%	4,824	2.71%
Location, n (%)				
Private residence	20,615	8.64%	16,810	9.43%
Non-private location	3,232	1.35%	2,293	1.29%
Trade or service	2,212	0.93%	1,403	0.79%
Sports/athletics	2,122	0.89%	615	0.35%
Industrial/construction	1,255	0.53%	962	0.54%

A total of 238,661 patients were included in the control and 178,224 in the study cohorts to describe injury characteristics. There was a general decrease in the majority of injury patterns in the study cohort (RR: 0.69-0.81, p < 0.001) (Table 2). The strongest percent change was seen for foot and ankle injuries (-1.63%). Mechanism of injury followed a similar trend with some exceptions. An increase in bicycle accidents (RR: 1.07, 95% CI: 1.04-1.11, p = 0.001) and assaults (RR: 1.17, 95% CI: 1.14-1.20, p < 0.001) was observed in the study cohort. A decrease in presentations was seen for the remainder of the categories. While motorcycle accidents did show a positive rate ratio, the 95% CI crossed 1. A decrease in suspected and confirmed physical abuse of adults and children was observed (RR: 0.57-0.76, p < 0.001) with a minimal percent change ranging from -0.08% to -0.02%.

**Table 2:** Percent change and rate ratio ^a^ Percent change is defined as a percent of the total for the study cohort (April-June 2020) minus the control cohort (April-June 2019).

	Percent change^a^	Rate ratio	95% CI	P-value
Demographics				
Male	1.78%	0.64	(0.63-0.65)	<0.001
Female	-1.56%	0.60	(0.59-0.61)	<0.001
Ethnicity				
Not Hispanic or Latino	1.01%	0.63	(0.62-0.64)	<0.001
Hispanic or Latino	-0.75%	0.56	(0.55-0.57)	<0.001
Race				
White	2.01%	0.64	(0.63-0.65)	<0.001
Black or African American	-1.56%	0.56	(0.55-0.57)	<0.001
Asian	-0.42%	0.51	(0.49-0.53)	<0.001
American Indian or Alaskan Native	0.02%	0.68	(0.61-0.76)	<0.001
Native Hawaiian or Pacific Islander	0.02%	0.67	(0.60-0.74)	<0.001
Characteristics				
Injury pattern				
Head	0.47%	0.75	(0.74-0.76)	<0.001
Neck	-0.48%	0.73	(0.72-0.74)	<0.001
Thorax	0.95%	0.78	(0.77-0.79)	<0.001
Abdomen/pelvic	0.72%	0.77	(0.76-0.78)	<0.001
Knee and lower leg	-0.34%	0.74	(0.73-0.75)	<0.001
Wrist, hand, fingers	-0.29%	0.74	(0.73-0.75)	<0.001
Ankle and foot	-1.63%	0.69	(0.68-0.70)	<0.001
Shoulder and upper arm	0.36%	0.76	(0.75-0.77)	<0.001
Elbow and forearm	0.71%	0.77	(0.76-0.79)	<0.001
Multiple trauma	0.43%	0.81	(0.78-0.83)	<0.001
Burns	0.34%	0.79	(0.77-0.81)	<0.001
Mechanism of injury				
Fall	-0.31%	0.74	(0.73-0.75)	<0.001
Car occupants	-1.71%	0.62	(0.61-0.64)	<0.001
Motorcycle accidents	0.51%	0.96	(0.92-1.00)	0.06
Pedestrian accidents	0.00%	0.75	(0.72-0.77)	<0.001
Bicycle accidents	1.05%	1.07	(1.04-1.11)	0.001
Intentional self-harm	0.27%	0.94	(0.89-0.99)	0.02
Assault	2.77%	1.17	(1.14-1.20)	<0.001
Physical abuse				
Adult, suspected	0.00%	0.76	(0.66-0.87)	<0.001
Adult, confirmed	-0.08%	0.59	(0.53-0.66)	<0.001
Child, suspected	0.00%	0.74	(0.63-0.87)	<0.001
Child, confirmed	-0.02%	0.57	(0.46-0.71)	<0.001
Alcohol associated, n (%)	0.76%	1.04	(1.00-1.08)	0.06
Location				
Private residence	0.79%	0.82	(0.80-0.83)	<0.001
Non-private location	-0.07%	0.71	(0.67-0.75)	<0.001
Trade or service	-0.14%	0.63	(0.59-0.68)	<0.001
Sports/athletics	-0.54%	0.29	(0.26-0.32)	<0.001
Industrial/construction	0.01%	0.77	(0.70-0.83)	<0.001

There was a trend toward increasing numbers of alcohol-related presentations (0.76%) in the study cohort (RR: 1.04, 95% CI: 1.00-1.08, p = 0.06), though this was not statistically significant. Injuries decreased across all locations (RR: 0.29-0.82, p < 0.001). The strongest decrease was seen at sports and athletic venues (RR: 0.29, 95% CI: 0.26-0.32, p < 0.01).

## Discussion

Overall, there was a decrease in case presentation for the majority of traumatic conditions. The overall case rate decreased by 122,912 (62%), which is in accordance with prior studies examining ED utilization [12,13]. An increase in the number of patients who were male and white was noted in the study cohort. Other studies have noted a trend toward increases in the proportion of male patients [14]. This was thought to be related to the amplification of their predilection for risk-taking, which inherently predisposes males to traumatic injury. Adherence to stay-at-home orders was stronger in urban areas, which would tend to protect ethnic minorities disproportionately [15]. While factors such as the aging of the population and improved safety technology in motor vehicles have attributed to variation in trauma epidemiology over time [16], this is unlikely to contribute significantly to year-to-year variation, which is examined in this study.

When examining general modes of transportation, significant differences were observed. Unsurprisingly, there was a decrease in the number of patients injured as car occupants by 1.71%. The overall lack of elective travel, business commuting, and general transportation likely accounts for the decreased volumes seen both in this study and described in the literature [4,8]. In the literature, some centers report a decrease of up to 75% [3]. An increase in bicycle accidents by 1.05% was noted in this study, likely attributable to the increased use of bicycles for outdoor independent exercise and recreation with similar trends demonstrated elsewhere [17].

A substantial decrease in the number of injuries that occurred at sports or athletic venues was observed. Many studies surrounding trends in orthopedic injuries during the pandemic demonstrate similar results with reduced rates of sports-related injuries [18], particularly in children [19], owing to the absence of organized sports. Interestingly, while overall occurrences decreased, one study reported that 31% of orthopedic injuries were related to a new hobby that was developed during a local lockdown [20]. While outdoor activities seem attractive as a socially distant means of exercise, they are not without its risk and standard precautions should be encouraged when undertaking new forms of physical fitness.

The overall increase in alcohol-associated injuries (0.77%), assaults (2.77%), and self-harm (0.27%) found in this study speak to perhaps one of the most underappreciated sources of morbidity during the COVID-19 pandemic. The most dramatic increase was seen in the number of cases where the assault was described as the mechanism of injury. Similar studies have noted steady rates of violent injuries despite overall decreased volumes [14] and increased cases of non-accidental penetrating trauma [21]. Not unique to the pandemic, studies have shown that rates of penetrating trauma increase during times of crisis [22].

This study demonstrated a modest increase in the number of cases that were alcohol-related. Other studies conducted in urban areas saw increased positivity in screening for substances of abuse by nearly 21% [4]. Alcohol use not only directly contributes to increased incidence of injury but also indirectly contributes to the exacerbation of mental health disorders, cases of abuse, and depression, potentially leading to intentional self-harm, which was also observed [23]. This highlights the concerning fact that substance abuse, mental health, and trauma are interconnected. A multifactorial public health approach is likely needed to combat mental health and substance use in times of high stress and anxiety to prevent not only traumatic injury but other long-term sequelae.

The issue of physical abuse of adults and children likely represents a dynamic issue. In this study, there was little difference seen in the cases of confirmed or suspected abuse of adults or children. Similar studies have shown mixed results. While some authors report an increased incidence of child abuse, others observed the opposite trend [24,25]. This issue is likely multifactorial and related somewhat to timing and unique social circumstances. Studies that examine abuse and intimate partner violence tend to show no difference [7] or decrease [26] in the immediate peri-lockdown phase (March through May 2020). This study seems to mirror these results. In one study, it was observed that cases of intimate partner violence increased later in the pandemic [27]. It has been hypothesized that multiple caregivers being present in the home during periods of lockdown is protective against non-accidental trauma in children [25]. Additionally, it seems that as tensions rise and financial hardship increased as the pandemic continued, the risk of interpersonal violence and abuse increased. This suggests that short periods of lockdown to curb the rapid local spread of disease may mitigate the effects of stressors that tend to develop over time.

Limitations

The most significant limitation of this study is its retrospective nature. Limitations to the utilization of large datasets such as TriNetX have been described including certain percentages of missing data and selection bias [28,29]. Indeed, the cohorts analyzed in this study were abbreviated samples of larger cohorts selected in a non-randomized fashion. The convenience sampling used to build cohorts could lead to sampling bias and may limit the broad generalization of the study. Missing information was demonstrated by significant percentages of gender, ethnicity, and race characteristics listed as unknown. Furthermore, the use of electronic health record data inherently involves the risk of incomplete or imprecise data entry when coding, which was the basis for reporting in this study.

## Conclusions

This study utilizes a large, population-level database to describe alterations in the patterns of traumatic injuries during COVID-19 pandemic-related stay-at-home initiatives. Due to its observational nature, it cannot claim the stay-at-home orders as the cause of the variation in traumatic injury. However, it does seem to mirror trends seen at local and regional levels and can serve to guide further inquiry, scholarly work, and policy development.

To many around the world who have personally experienced some variation of stay-at-home mandates, the changes in injuries related to transportation and recreation are probably unsurprising. However, this study highlights a critical issue pertaining to the complex interaction between pandemic-related violence, substance abuse, and self-harm. This likely represents a key area of public health preparedness during subsequent crises, pandemic or otherwise. Such initiatives will likely have a direct and indirect effect on the burden of traumatic injuries observed.
